# Thyroid Artery Embolization as Salvage Therapy in Recurrent Papillary Thyroid Carcinoma: A Case Report

**DOI:** 10.1159/cro/abzag047

**Published:** 2026-09-08

**Authors:** Junjie Hao, Hao Jiang, Honghua Sun

**Affiliations:** Oncology Department, The Affiliated Hospital of Yanbian University, Yanji, China

**Keywords:** Thyroid cancer, Interventional therapy, Unconventional treatment, Significant efficacy

## Abstract

**Introduction:**

One case involved a 55-year-old male who was admitted to hospital with thyroid cancer.

**Case Presentation:**

Chose interventional therapy (an unconventional treatment) after the conventional treatment failed.

**Conclusion:**

Eventually, the thyroid mass significantly decreased and achieved effective treatment.

## Introduction

This case report has been reported in line with the CARE criteria. The conventional treatment methods for papillary thyroid carcinoma include surgical treatment (surgical treatment, lymph node dissection, etc.), ^131^I therapy, endocrine therapy, radiotherapy, thermal ablation therapy, systemic treatment, etc. This case demonstrates the significant efficacy of using an unconventional method – interventional therapy to treat a patient with papillary thyroid carcinoma.

## Case Presentation

A 55-year-old male, who had undergone thyroid cancer surgery 10 years ago and whose neck mass was discovered 5 years ago, was admitted to the oncology department for treatment. Ten years ago, he was treated at the Second Hospital of Jilin University for neck masses and was diagnosed with papillary thyroid carcinoma. He underwent radical thyroidectomy + left cervical dissection. Nine years ago, a new mass was found in the left neck, and he went to the Second Hospital of Jilin University for treatment again. Considering tumor recurrence with multiple metastases, surgical treatment was not recommended. So he received two cycles of radiotherapy. Eight years ago, due to the progressive enlargement of the mass, he visited Beijing Hospital and underwent bilateral thyroid cancer surgery, and was given tracheal intubation. Five years ago, a right neck mass continued to grow, and no systemic treatment was given. Our physical examination revealed that the patient’s general condition was poor. A huge mass was seen in the right neck, with ulceration and bleeding (Fig. 1a). His white cell count, C-reactive protein, interleukin-6, and fungal D-glucan levels were all within normal limits (4.1 × 10^9^/L, 0.68 mg/L, 2.73 pg/mL, <10 pg/mL, respectively), but his hemoglobin was abnormal (52 g/L). Total triiodothyronine, total thyroxine, free triiodothyronine and free thyroxine was decreased, the values were 0.49 ng/mL, 3.56 μg/dL, 1.39 pg/mL, and 0.55 ng/dL, respectively. And thyroid-stimulating hormone was increased (13.70 uIU/mL), thyroglobulin antibody was increased (389.00 IU/ML). Other tests (coagulation function, liver function, kidney function, urine routine tests, etc.) were all normal. Tumor markers in patients’ serum were all negative. The computed tomography angiography of the thyroid arteries showed that there were large, irregular-shaped masses in both thyroid glands, protruding outward from the neck. The lesions showed heterogeneous enhancement. The right lesion was larger, approximately 13.2 cm × 10.4 cm in size (Fig. 2a). Part of the lesion had liquefied. The right superior and inferior thyroid arteries were compressed and pushed outward, and the boundaries with the right common carotid artery and internal carotid artery were still clear. The left lesion showed more significant enhancement compared to the right, with an infiltration growth of approximately 8.2 cm × 6.6 cm (Fig.2a). The boundaries were not clear, and the lesion had unclear boundaries with the internal carotid artery and common carotid artery. Multiple enlarged lymph node shadows could be seen in the bilateral necks, and some of them growed in fusion with the lesions. Multiple sized masses could be seen in both lungs. Histological examination of the neck mass (Fig. 3) revealed papillary thyroid carcinoma. The results of the immunohistochemical analysis were as follows: TG(+), Galectin-3(+), CD56(−).

**Fig. 1.**
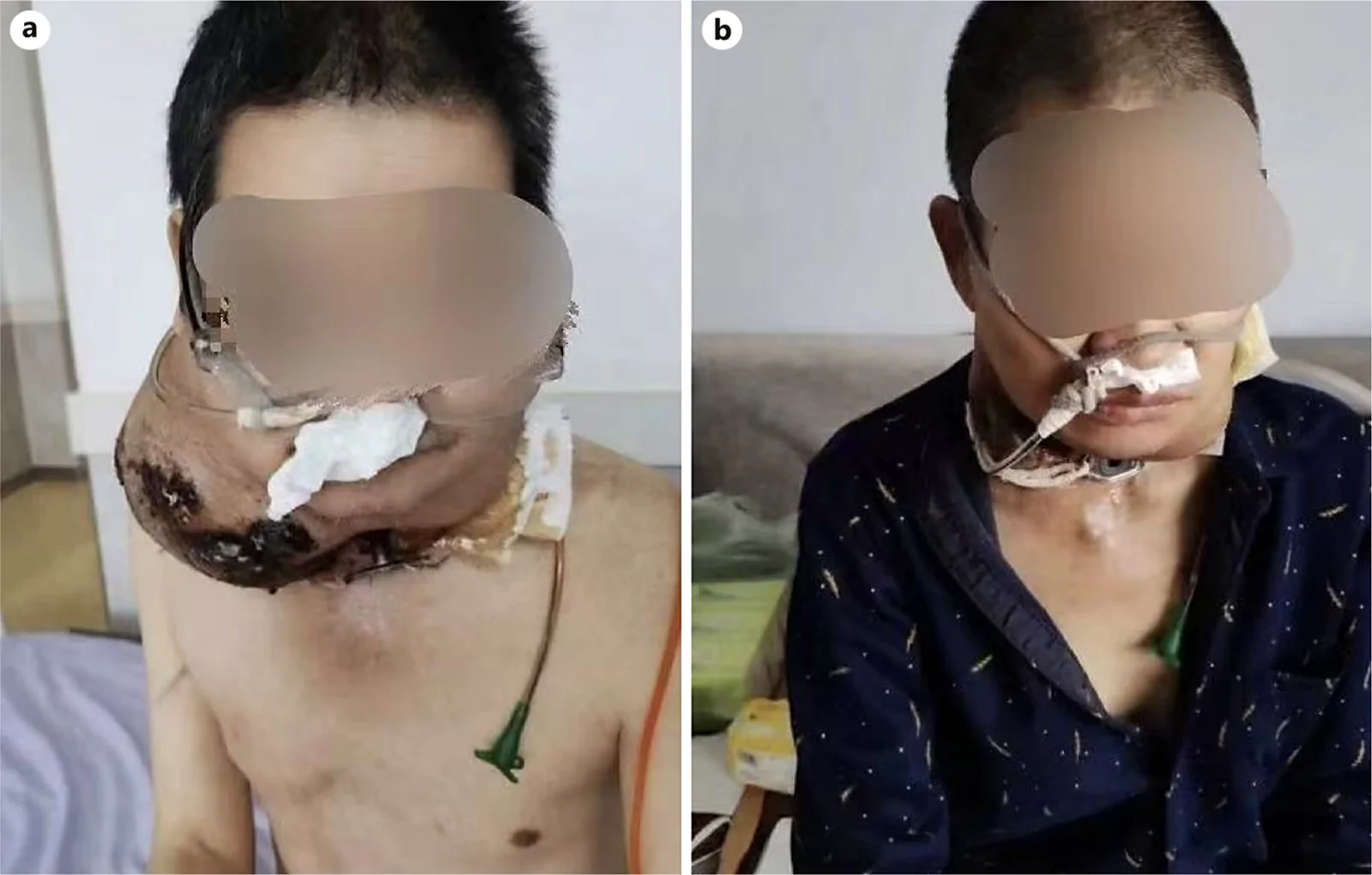
The physical examination revealed that a huge mass was seen in the right neck, with ulceration and bleeding before the treatment (**a**) and the neck mass had significantly shrunk after the fourth treatment (**b**).

**Fig. 2.**
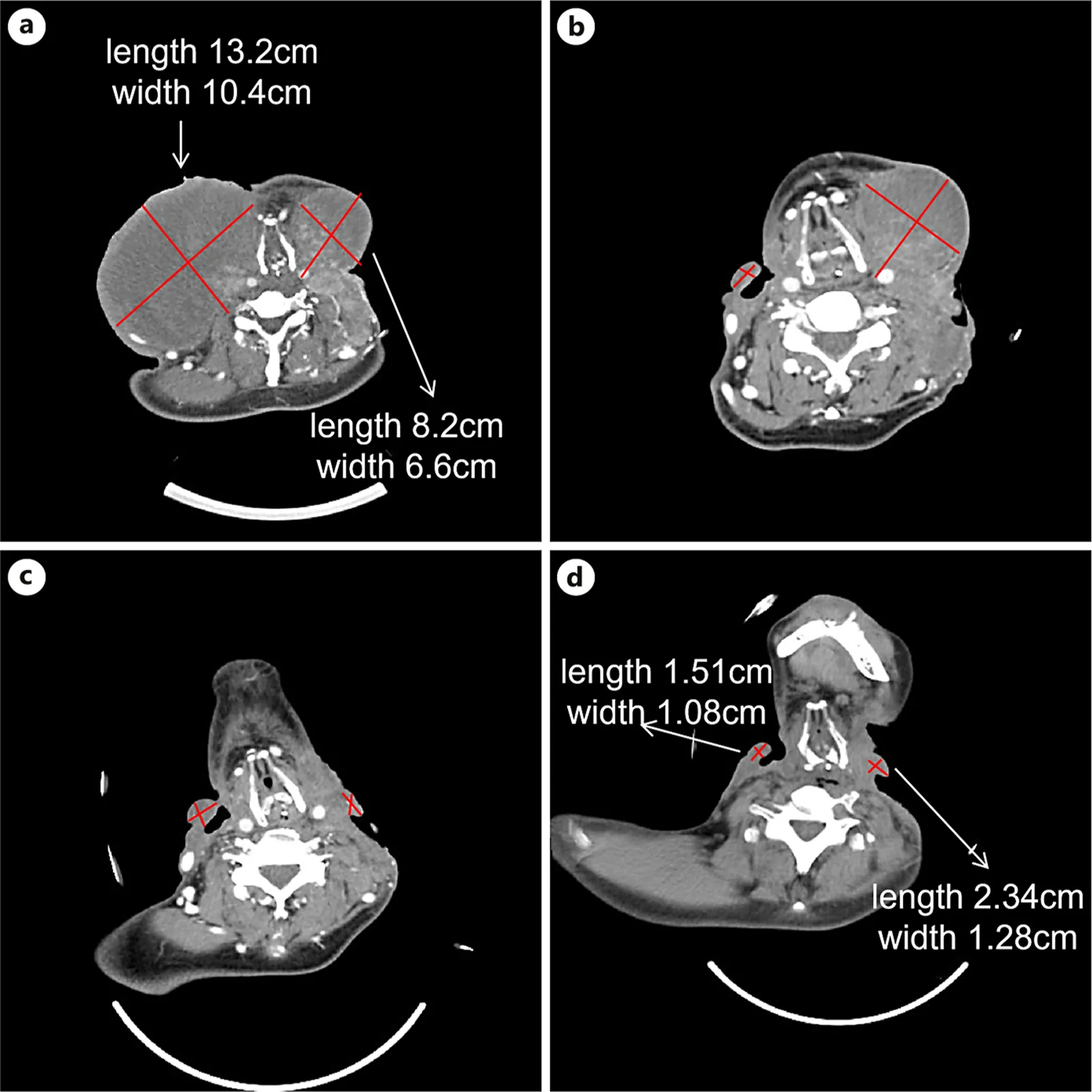
The CTA of the thyroid arteries showed that an occupying lesion (length 13.2 cm, width 10.4 cm) in the right thyroid gland, a mass(length 8.2 cm, width 6.6 cm) in the left thyroid gland (**a**) before the treatment. A computed tomography (CT) scan of the thyroid showed that the lesions reduced after the second (**b**) and third treatment (**c**). A CT scan of the thyroid showed that the lesions significantly reduced (the right occupying from 13.2 cm × 10.4 cm to 1.51 cm × 1.08 cm; the left occupying from 8.2 cm × 6.6 cm to 2.34 cm × 1.28 cm) after the fourth treatment (**d**). CTA, computed tomography angiography.

**Fig. 3.**
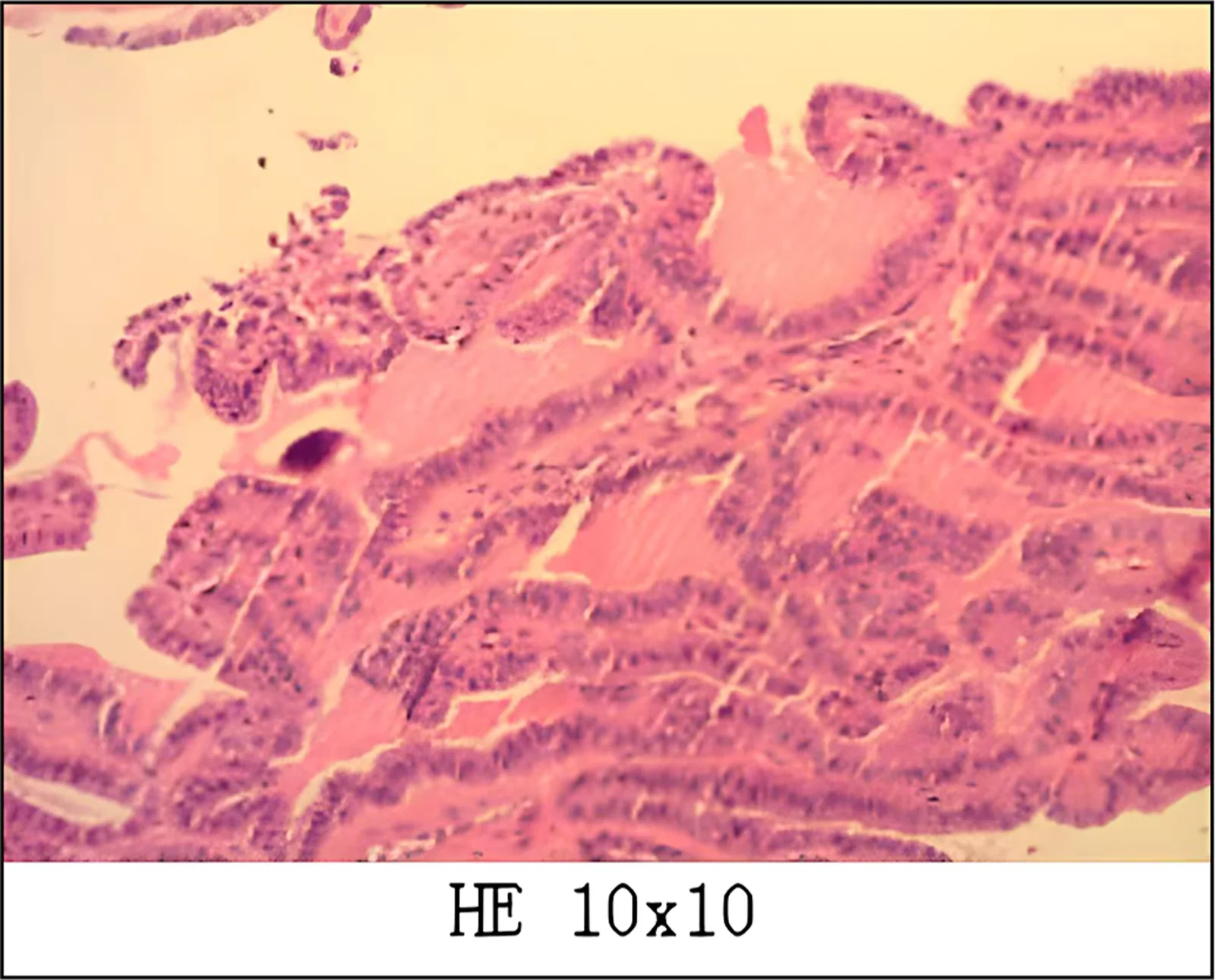
Histological examination revealed papillary thyroid carcinoma. The results of the immunohistochemical analysis were as follows: TG(+), Galectin-3(+), CD56(−).

A diagnosis of papillary thyroid carcinoma was made, and the clinical stage was T_4a_N_1b_M_1_, stage III. The conventional treatment methods [1] for papillary thyroid carcinoma include surgical treatment (surgical treatment, lymph node dissection, etc.), ^131^I therapy, endocrine therapy, radiotherapy, thermal ablation therapy, systemic treatment, etc. [2]. Given the poor condition of the patient, who has undergone surgical treatment and radiotherapy but with poor treatment outcomes and repeated recurrences, even with metastasis, these two treatment options were not considered. The patient had a large neck mass, and ^131^I was difficult to effectively accumulate, possibly due to insufficient radiation dose, resulting in treatment failure and an increased risk of systemic side effects; thermal ablation had limited conductivity, which might lead to incomplete ablation and easy recurrence of residual cancer cells, and these two treatments were not recommended either. The patient’s neck mass was large, compressing the trachea and severely affecting respiratory function, so a single endocrine therapy or systemic therapy had limited effect and was difficult to solve the problem in time. Therefore, we did not advocate using the conventional treatment methods. However, considering the patient’s poor condition, large neck mass, and the presence of compression, treatment must be carried out as soon as possible. Eventually, we chose an unconventional treatment method – interventional therapy (thyroid artery embolization therapy). Within 5 months, we performed four thyroid artery embolization treatments on the patient. All procedures were performed via the Seldinger technique through the right femoral artery approach under local anesthesia. Diagnostic angiography was initially performed to identify the feeding arteries. In this case, a total of 7 feeding arteries were identified and treated over the four sessions, including the bilateral superior thyroid arteries and inferior thyroid arteries, as well as collateral branches from the thyrocervical trunk and costocervical trunk. A microcatheter was advanced coaxially into the target arteries as distally as possible to avoid nontarget embolization. Polyvinyl alcohol particles (300–500 μm) mixed with contrast medium served as the embolic agent during the four sessions of thyroid artery embolization. The embolization endpoint was defined as near-stasis of blood flow within the feeding arteries and disappearance of tumor blush, with patency of the external carotid artery maintained. Patients were monitored for vital signs throughout the procedure. Post-embolization syndrome (fever, pain, nausea) was managed symptomatically with analgesics and antipyretics. Prophylactic antibiotics were administered to prevent infection of the necrotic tissue. By blocking the blood supply to the tumor through blocking the blood supply arteries, the tumor was deprived of blood and oxygen, thereby inhibiting tumor growth. To improve the treatment effect, lenvatinib targeted therapy was also given. Lenvatinib was initiated at a dose of 8 mg once daily, starting on the day of the first embolization procedure. The treatment was continued concurrently with the interventional therapy sessions and was ongoing at the time of the last follow-up. Throughout the treatment course, no dose reductions or interruptions were required. The physical examination showed that the general condition was good, and the neck mass had significantly shrunk. The treatment is effective: the physical examination showed that the general condition was good, and the neck mass had significantly shrunk (Fig. 1b). And the hemoglobin level rose to 67 g/L. Total Triiodothyronine was still at a lower level before (0.76 ng/mL), but total thyroxine, free triiodothyronine, free thyroxine, thyroid-stimulating hormone and thyroglobulin antibody have returned to normal levels. A CT scan (Fig. 2d) of the thyroid showed that the lesions reduced (the right occupying from 13.2 cm × 10.4 cm to 1.51 cm × 1.08 cm; the left occupying from 8.2 cm × 6.6 cm to 2.34 cm × 1.28 cm). The angiographic images before and after each of the four interventions are shown in Figure 4. The changes in key laboratory indicators, including thyroglobulin antibody, thyroid-stimulating hormone, total triiodothyronine, and total thyroxine, are illustrated in Figure 5.

**Fig. 4.**
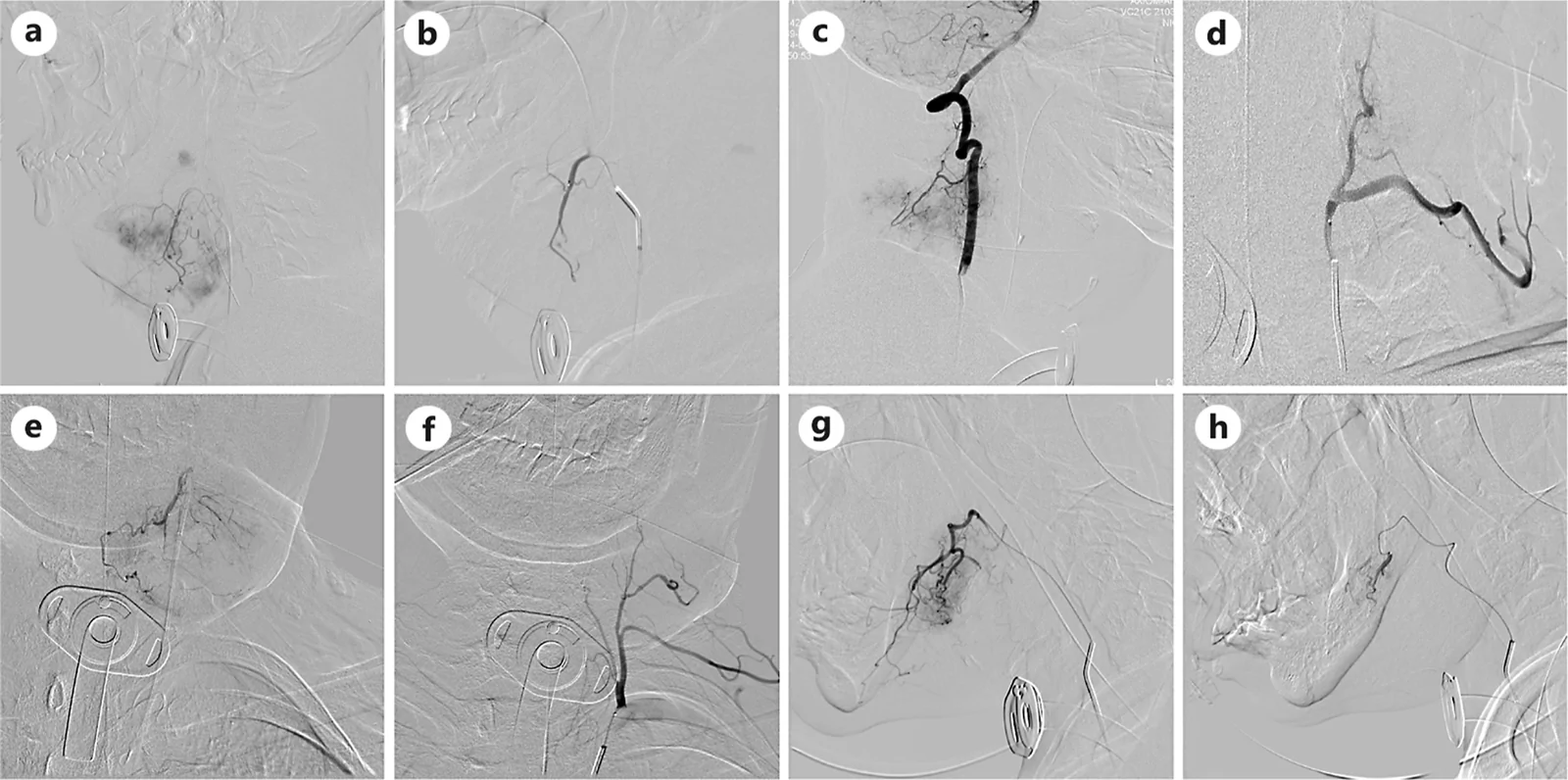
**a**, **b** Represent the pictures before and after the first intervention treatment. **c**, **d** Represent the pictures before and after the second intervention treatment. **e**, **f** Represent the pictures before and after the third intervention treatment. **g**, **h** Represent the pictures before and after the fourth intervention treatment.

**Fig. 5.**
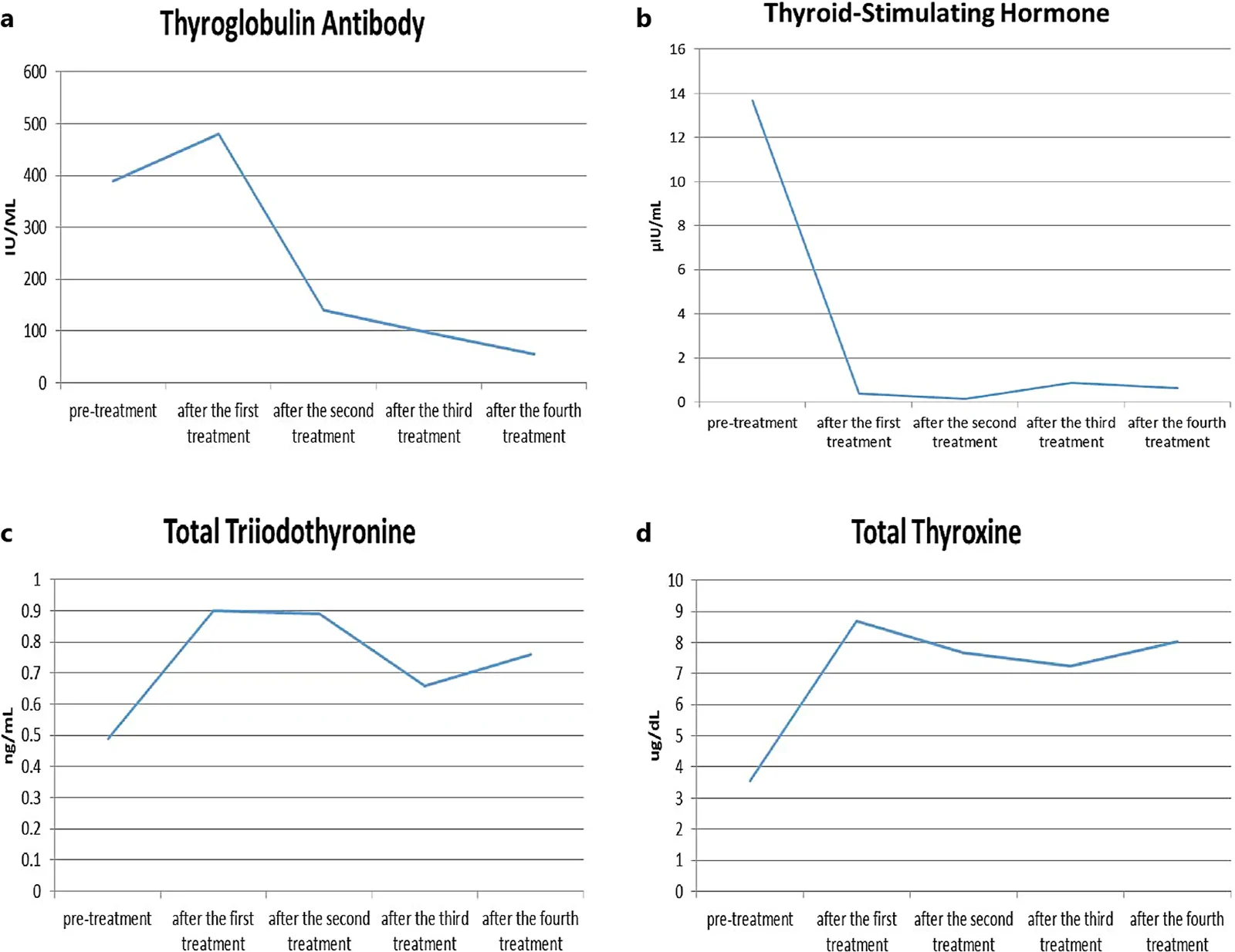
**a** Shows the changes in the values of thyroglobulin antibody before treatment and after four treatments in the form of a line graph. **b** Shows the changes in the values of thyroid-stimulating hormone before treatment and after four treatments in the form of a line graph. **c** Shows the changes in the values of total triiodothyronine before treatment and after four treatments in the form of a line graph. **d** Shows the changes in the values of total thyroxine before treatment and after four treatments in the form of a line graph.

## Postoperative Complications and Follow-Up

All four sessions of thyroid artery embolization were technically successful. The patient experienced expected post-embolization syndrome, characterized by mild to moderate neck pain and low-grade fever (<38.5°C) within 48 h after each procedure. These symptoms were effectively managed with standard analgesics and antipyretics. No severe complications, such as nontarget embolization leading to skin necrosis, nerve injury, or stroke, were observed. Furthermore, the pre-existing ulcerated neck mass did not show signs of aggravated infection following the procedures. The patient was followed up for 6 months after the completion of the fourth intervention. During this period, the significant reduction in the neck mass was sustained. The patient’s respiratory function remained stable, and no new distant metastases were detected. The patient reported a substantial improvement in quality of life, including relieved compression symptoms and reduced care burden for the neck ulcer.

## Discussion and Conclusions

Thyroid artery embolization therapy has the advantages of low trauma, quick recovery, accurate positioning, and strong repeatability, and is applicable to the treatment of various diseases such as tumors and vascular disorders. However, in clinical practice, thyroid artery embolization is mostly used for the treatment of hyperthyroidism, and there are few literature reports on its use in treating thyroid cancer. This case report provides us with a new idea: for patients with recurrent thyroid cancer who do not respond well to conventional treatment, if the indications are met and there are no contraindications, thyroid artery embolization therapy can be considered. However, it is important to acknowledge that the patient in this case received concurrent treatment with Lenvatinib, a multi-kinase inhibitor, alongside the interventional procedures. Lenvatinib has demonstrated significant efficacy in radioactive iodine-refractory differentiated thyroid cancer by inhibiting tumor angiogenesis and growth through the targeting of VEGFR and other pro-oncogenic kinases. In this case, the rapid and substantial reduction of the tumor mass and the prompt relief of compression symptoms immediately following the embolization procedures strongly suggest that thyroid artery embolization played a dominant role in the initial, local tumor control by physically cutting off the blood supply. However, the systemic effect of Lenvatinib likely contributed to controlling potential microscopic metastases and inhibiting the recanalization of embolized vessels. Therefore, the remarkable efficacy observed is likely attributable to the synergistic effect of local devascularization and systemic targeted therapy, rather than the embolization alone. Regarding whether this therapy is universal, how the recurrence rate is affected, and whether it can achieve better therapeutic effect when combined with other treatments, more large-sample, multi-center clinical studies are needed. Recently, there has been continuous progress in the molecular knowledge of thyroid cancer, which not only improves the diagnostic level of thyroid cancer [3] but also promotes the formulation and exploration of innovative drug treatment plans [4], providing personalized treatment options for some patients with severe conditions. Exploring potential new molecular therapeutic targets and developing personalized clinical management methods have also provided new ideas for the treatment of thyroid cancer [5].

## Statement of Ethics

This study was performed in accordance with the Declaration of Helsinki. This human study was approved by The Science and Technology Ethics Review Committee of the Affiliated Hospital of Yanbian University (Yanbian Hospital) – approval: December 31, 2025. All adult participants provided written informed consent to participate in this study. Written informed consent was obtained from the individual(s) for publication of the details of their medical case and any accompanying images. The CARE Checklist for this case report is available as online supplementary material (for all online suppl. material, see https://doi.org/10.1159/cro/abzag047).

## Conflict of Interest Statement

The authors declare that they have no competing interests.

## Funding Sources

No funding was used in this study.

## Author Contributions

J.H.: conception and design of study, acquisition of data, data analysis and interpretation, drafting of manuscript, and approval of final version of manuscript. H.J.: acquisition of data, data analysis, and approval of final version of manuscript. H.S.: ideals, conception and design of study, and approval of final version of manuscript.

## Data Availability

The data that support the findings of this study are not publicly available due to privacy reasons, but are available from the corresponding author upon request.
